# Reliability, validity and critical appraisal of the cross-cultural adapted German version of the Mayo Elbow Performance Score (MEPS-G)

**DOI:** 10.1186/s13018-022-03210-5

**Published:** 2022-06-25

**Authors:** A. Papen, T. Schöttker-Königer, A. Schäfer, F. Morrison, B. Hollinger, K. J. Burkhart, R. Nietschke, A. Zimmerer, N. Maffulli, F. Migliorini, Marco M. Schneider

**Affiliations:** 1 Faculty of Social Work and Health, University of Applied Science and Art (HAWK), Hildesheim, Germany; 2 German Association for Manual Therapy (DVMT e.V.), Dresden, Germany; 3 Centre for Sports Orthopedics and Special Joint Surgery, Orthopedic Hospital Markgroeningen, Markgroeningen, Germany; 4 Arcus Sportklinik, Pforzheim, Germany; 5 University of Cologne, Cologne, Germany; 6 Orthio Praxisklinik, Karlsruhe, Germany; 7 Department of Medicine, Surgery and Dentistry, University of Salerno, Baronissi, Italy; 8 School of Pharmacy and Bioengineering, Keele University School of Medicine, Thornburrow Drive, Stoke on Trent, UK; 9 Centre for Sports and Exercise Medicine, Barts and The London School of Medicine and Dentistry, Queen Mary University of London, London, UK; 10 Department of Orthopedics, Trauma and Reconstructive Surgery, RWTH Aachen University Hospital, Aachen, Germany; 11 University of Witten/Herdecke, Witten, Germany

**Keywords:** Elbow joint, MEPS, Patient-related outcome measures, Reliability, Validity

## Abstract

**Background:**

The Mayo Elbow Performance Score (MEPS) is a rating system consisting of four dimensions to evaluate elbow performance. It is a common tool for assessment of elbow impairments worldwide. We determined the validity and reliability of its German version (MEPS-G) after cross-cultural adaptation.

**Methods:**

Six investigators examined 57 patients with elbow pathologies. The MEPS-G was compared to validated elbow scores such as the German versions of DASH, the Oxford Elbow Score, pain level and subjective elbow performance on a VAS. Inter-rater reliability (IRR) and validity of the score and its dimensions were also reviewed. Verification was performed using the intraclass correlation coefficient (ICC), the prevalence and bias with adjusted Kappa (PABAK) and the Spearman correlation.

**Results:**

The IRR of the MEPS-G score was moderate (ICC (2.1) = 0.65). The IRR of the four individual dimensions was moderate to high (*K*_PABAK_ = 0.55 -0.81). Validity for the sum score (*r* = 0.52–0.65) and the dimensions pain (*r* = 0.53–0.62), range of motion (*r* = 0.7) and stability (*r* = − 0.61) was verified. The function subscale reached insufficient validity (*r* = 0.15–0.39).

**Conclusion:**

The MEPS-G is not sufficiently valid, which is consistent with its English version. The patient-based dimensions were a weakness, demonstrating high risk of bias. There is no general recommendation for the utilization of the MEPS-G as outcome measurement for patients with elbow pathologies.

## Introduction

The increasing understanding of elbow anatomy, advances in surgical techniques and their availability led to an expansion of elbow procedures over the last few years [1–5]. Outcomes of surgical and non-surgical treatment should be evaluated with patient-related outcome measures (PROMs), which detail patient perception on management, health and quality of life [6, 7]. PROMs can be divided into generic, joint-specific or disease-specific questionnaires [8]. PROMs are more frequently utilized for research purposes, become increasingly valuable in health economics [9, 10] and may improve physician–patient relations [10–12]. The physician-administered Mayo Elbow Performance Score (MEPS), also known as the Mayo Elbow Performance Index [13], is currently the most commonly used outcome measure of elbow impairments in clinical trials worldwide [14]. Despite the broad international utilization of the MEPS [14–19], it is described as not being adequately validated [25] and only a relatively small number of translations, and cross-cultural adaptations have been performed.

The present study determined the validity and reliability of the German version of the MEPS (MEPS-G) after cross-cultural adaptation and gives a critical appraisal of its psychometric properties and those of the MEPS itself.

## Methods

The German translation and cultural adaptation of the MEPS were completed according to the steps by Beaton et al. [20] using a “translation–back translation” method. A pilot testing of the pre-final MEPS-G with German-speaking subjects was performed on 57 participants, to confirm the comprehensibility and to search for possible problems with data collection or examination. Apart from minor adjustments, the MEPS was converted into the German version MEPS-G without difficulty [21].

Since the MEPS-G consists of four individual dimensions, we evaluated its total score as well as the four dimensions for reliability and validity in a cross-sectional survey.

For validation, instability was measured with the combination of various tests (Table 1) and range of motion (ROM) was quantified by the use of an electric goniometer (Easyangle, Meloq AB, Stockholm, Sweden). Furthermore, pain was checked for criterion validated with comparison to pain on a visual analogue scale (VAS) as well as the pain dimension of the German Oxford Elbow Score (OEB). The dimension function of the MEPS-G was compared to the Disabilities of the Arm, Shoulder and Hand adapted to German (DASH-G), to the dimension function of the OEB as well as function scale derived from the dimension function of the MEPS-G. The DASH-G, the OEB and the elbow performance using VAS were additionally used to validate the total score of the MEPS-G.

**Table 1:** Test battery for measuring elbow instability

Type of instability	Assessment	Diagnostic accuracy
Posterolateral rotatory instability (PLRI)	Chair push-up test Prone push-up test	Sens. 88% Sens. 88%
Valgus instability	Valgus stress test Moving valgus stress test	Pain: Sens. 65%/ Spec. 50%; Laxity: Sens. 19%/Spec. 100% Sens. 100%/Spec. 75%
Varus posteromedial rotatory instability (VPMI)	Gravity-assisted varus stress test	No data

### Instruments

#### Mayo Elbow Performance Score

**Fig. 1:**
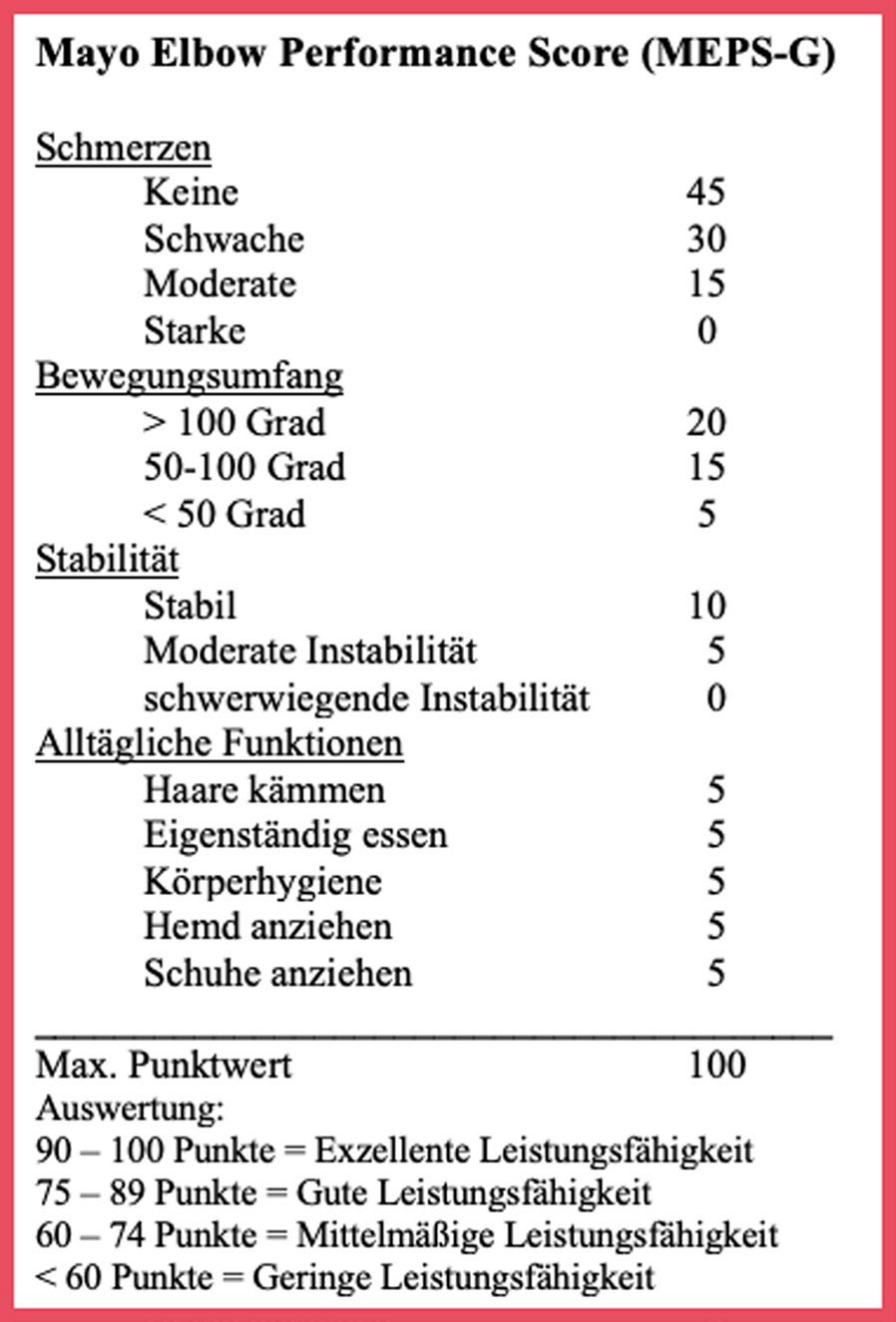
German version of the MEPS

The MEPS [22] is a multi-dimensional assessment tool to evaluate elbow performance. It combines the clinically measurable dimensions of mobility and stability with the subjective patient-based aspects of pain and function in one index. The total score is calculated from the score in each of the four dimensions (Fig. 1) and indicates the latent construct of overall elbow performance. No manuscript or instructions for handling have been conceptualized, so that the implementation is ultimately left to each investigator. It was developed without critical methodical criteria [23, 24] and described as not being adequately validated [25].

#### Test battery for measuring elbow instability

A generally valid assessment set of elbow instabilities is not yet available. Among other reasons, there are no generally accepted rules for the classification of elbow instability [13]. The MEPS-G does not contain any information on the assessment of elbow stability. For this reason, this study used a combination of tests for the evaluation of each form of elbow instability (see Table 1). The selection of test procedures was based on the review by Karbach and Elfar [26] and Zwerus et al. [27]. The investigators rated the tests on the basis of a binary evaluation scheme “test positive” or “test negative.” The number of positive tests was added up to a total score.

#### Elbow Performance on a Visual Analogue Scale

The VAS on elbow performance was used in a similar way by Turchin et al. [28] to measure the latent construct of elbow performance. The author of this study devised the VAS to assess the subjective elbow performance by both the investigator and the patient. The goal was to compare both views on elbow performance and to assess the extent, to which the perceived performance matches the MEPS total score.

In this study, the continuous VAS consisted of a ten-centimeter-long horizontal line, with zero centimeters indicating the “lowest possible elbow performance” and ten centimeters indicating “excellent performance,” corresponding to the MEPS classifications [22].

#### MEPS-G Function Scale

The dimension function is operationalized with a binary scheme (0 or 5 points). For a more detailed validation of the dimension, a 5-point Likert scale based on the functions queried in MEPS-G was used. This 5-point scale was filled in by the patients as well as by an investigator (Fig. 4). The aim of the scale was to assess the extent to which the binary dimension function of the MEPS-G is able to represent the patients’ and investigators’ perspective.

#### Disabilities of the arm, shoulder and hand

The questionnaire is an instrument that measures the functional ability or impairment of the upper extremity [29] and is one of the most frequently validated assessment tools [30]. Its German translation, the DASH-G, achieved similar psychometric properties as the American original [31], is frequently used in German-speaking countries and was therefore considered suitable for comparison. The DASH was used in the validation study of Turchin et al. [28] and thus allows a comparison with other MEPS studies.

#### Oxford Ellenbogen Bewertung

The “Oxford Ellenbogen Bewertung” (OEB) is the German translation of the Oxford Elbow Score (OES) [32]. The OES is used to classify elbow complaints and to assess the success of a medical intervention of the elbow [33]. The strengths of the OES reside in the representation of the patients’ perspective, the specification on elbow complaints and the high sensitivity to change [33], and its overall methodological and psychometric qualities [25].

#### Pain on a Visual Analogue Scale

The Visual Analogue Scale Pain is the most commonly used measurement tool of pain in both research and practice [23]. In this study, VAS-Pain was used to measure the “current pain of the elbow joint.” The minimal and maximal pain were referred to as “no pain” and “worst imaginable pain.”

### Participants and data collection

All patients presented in elbow clinics between October 1, 2018, and November 31, 2018, were invited to participate to this study. Only patients with elbow complaints of different nature and agreed to participate in the study were included. All patients were reviewed for sufficient linguistic and cognitive abilities to understand the instructions and fill in the questionnaires. Patients were excluded if they perceived any pain radiating from the proximal side into the elbow joint or did not consent or did not meet the inclusion criteria in any way.

**Fig. 2:**
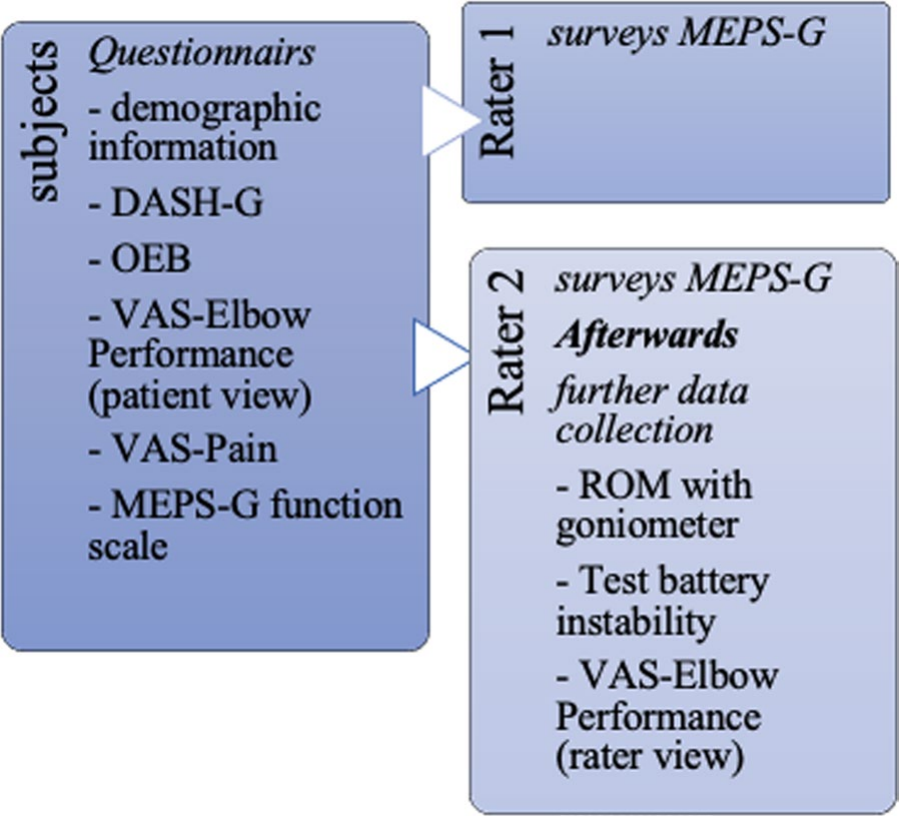
Visualized representation of data collection. Abbreviations: DASH-G = German Version of Disabilities of the Arm, Shoulder and Hand: OEB = German Version of the Oxford Elbow Score; ROM = range of motion; VAS = visual analogue scale

In a cross-sectional survey, six examiners, five male specialists in orthopedics and trauma surgery (professional experience: 9 years ± 4.3) and one female physiotherapist (5 years), examined 57 patients with elbow complaints using the MEPS-G and the above-mentioned instruments for reasons of comparability. Two investigators who were paired differently each day were available for each day of data collection. All dimensions of the MEPS-G were surveyed by the six investigators. In the clinical setting, demographic data, DASH-G, OEB, VAS-Pain and VAS-Elbow Performance, and the MEPS-G functional scale were completed by all patients. The first survey of the MEPS-G was then carried out by Rater 1 (R1). Directly afterwards, Rater 2 (R2), who was blinded to the findings of the initial examination, ascertained the MEPS-G and the comparative instruments without the presence of R1 (Fig. 2).

### Statistical analysis

Data analysis was carried out using the statistical program STATA 14 (StataCorp LLC, College Station, Texas). The required sample size followed the recommendations of Giraudeau and Mary [34]. About 50 patients are required to determine the kappa value and to provide a reasonable number of dots in a Bland and Altman plot to estimate the limits of agreement.

To determine the inter-rater reliability of the metrically scaled variables, the intraclass correlation coefficient (ICC type 2.1) and the Bland–Altman method were used for the total score [35]. In addition, the measurement error (SEM) was calculated from the root of the error variance and the minimum detectable change (${\text{MDC}} = 1.96 \cdot \sqrt 2 \times {\text{SEM}}$) [36]. The inter-rater reliability of the ordinally scaled dimensions was calculated by means of the prevalence and bias-adjusted Kappa (PABAK) and percentage agreement [37].

Spearman correlation was used to determine the criterion validity and construct validity of the overall score and the individual dimensions of the MEPS-G. For the interpretation of correlation coefficients, the classification of Portney and Watkins [38] was applied (Table 2).

**Table 2:** Interpretation of correlation coefficient according to Portney and Watkins [38]

Amount of the coefficient	Interpretation
0–0.25	Little or no relationship
0.25–0.5	Fair relationship
0.5–0.75	Moderate-to-good relationship
> 0.75	Good-to-excellent relationship

### Ethical declaration

Ethics committee approval was granted by the ethics committee of the Landesärztekammer Baden-Württemberg, Stuttgart, Germany. The study was performed following the ethical standards in the 1964 Declaration of Helsinki. Written informed consent was obtained from each patient prior to participation.

## Results

### Sample

Fifty-seven subjects with elbow complaints were examined with the MEPS-G and the comparison instruments. The average age of the subjects (*n* = 57) was 47.5 years (±15.5; CI 95%: 42.9; 52), 43.5% were female. 65.9% of subjects had previously been operated on the elbow and 55.8% of them performed a hand-straining activity. The sample includes various pathologies, such as medial or lateral epicondylitis, osteoarthritis, elbow dislocations and fractures. The mean value of the MEPS-G score in this sample was 66.3 (MD 19.5; CI 95%: 61.1; 71.5) out of a maximum of 100 points. This corresponds to the MEPS-G assessment category of fair performance. The results of each dimension and the comparative instruments are presented in Table 3.

**Table 3:** Descriptive representation of the elbow performance according to the MEPS-G and the comparatives instruments (*n* = 57)

Characteristics	Patients (*n* = 57)	
MEPS-G (R2) (*n* = 57)	*Total score (0–100 points)* 66.3 ± 19.5 (CI 95%: 61.1; 71.5) Minimum: 30/Maximum: 100 → corresponds to classification “fair” elbow performance	
	*Pain (up to 45 points):* None: 10.5% Mild: 35.1% Moderate: 29.8% Severe: 24.6%	*Motion (up to 20 points):* Arc > 100°: 64.9% Arc 50–100°: 28.1% Arc < 50°: 7%
	*Stability (up to 10 points)* Stable: 68.4% Moderate instability: 21.1% Gross instability: 10.5%	*Function (yes/ no)* Comb hair: 82.5%/17.5% Feed: 80.7%/ 19.3% Hygiene: 84.2%/ 15.8% Shirt: 86%/14% Shoe: 89.5%/10.5%
		*Sum score function (0–25)* Median = 25; $I_{25}$ = 20; $I_{75}$ = 25
VAS-Elbow performance (patient)	*Specification in mm (0–100)* 45.7 ± 22.2 (CI 95%: 39; 52.5) Minimum: 0 / Maximum: 83 → *low to moderate elbow performance*	
VAS-Elbow performance (R2)	*Specification in mm (0–100)* 54.5 ± 26.3 (CI 95%: 46.9; 62.2) Minimum: 10/Maximum: 100 → *moderate elbow performance*	
MEPS-G Function Scale (patient)	*Total score (5–25)* Median = 10; $I_{25}$ = 6.25; $I_{75}$ = 15 → *moderate to good elbow performance*	

*MEPS-G* German Version of Mayo Elbow Performance Index; *R2* Rater 2; *VAS* visual analogue scale

### Testing of reliability

The inter-rater reliability of the total score of the MEPS-G revealed an ICC (2.1) of 0.65 (CI 95% 0.46; 0.78). To test the inter-rater reliability of a more homogeneous group, the physiotherapist was removed from the calculation. Excluding the physiotherapist as investigator, the inter-rater reliability increased (ICC (2.1) = 0.82; *n* = 28).

**Fig. 3:**
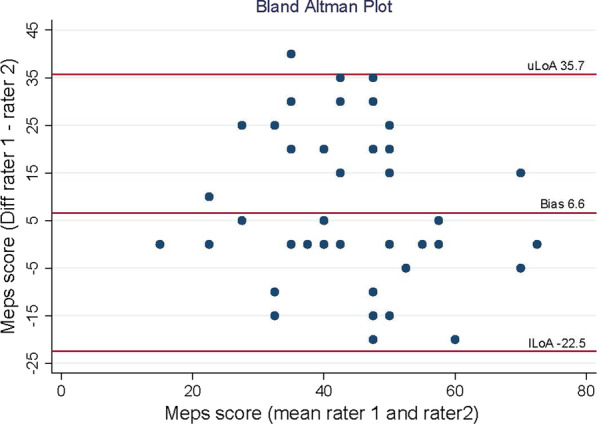
Bland–Altman Plot. The distribution of the absolute differences between rater 1 and rater 2 in relation to the mean of the two sum scores Abbreviations: Diff = difference

Table 4 presents the results of the inter-rater reliability. Figure 3 shows the Bland–Altman plot. The rather even distribution indicates that there are no systematic differences between Rater 1 and Rater 2 (Table 5).

**Table 4:** Inter-rater reliability of the total score of the MEPS-G

Statistical methodology	Parameter	Values (*n* = 57)
Intraclass correlation coefficient	ICC (2.1); 95% CI; P	0.65; 0.46; 0.78; *p* ≤ 0.001
Standard error (SEM)	$\sigma_{e}$	11.42
Bland–Altman method	Absolute bias	6.61
	Lower limits of agreement	− 22.53
	Upper limits of agreement	35.75

**Table 5:** Inter-rater-reliability of the MEPS-G dimensions (*n* = 57; *p* ≤ 0.001)

Endpoint	Pain	Motion	Stability	Function
PABAK (95% CI)	*κ* = 0.55 (0.4; 0.7)	*κ* = 0.81 (0.69; 0.93)	*κ* = 0.78 (0.64; 0.92)	*κ* = 0.75 (0.62; 0.88)
% agreement (95% CI)	83.1 (76.8; 89.5)	92.2 (86.4; 98.1)	90.2 (83.4; 96.9)	90.3 (84.6; 96.1)

*PABAK* prevalence-adjusted bias-adjusted kappa; *CI* confidence interval

The descriptive presentation of the MEPS-G dimensions showed an uneven distribution of the characteristics of the dimensions (Table 3). This is why the PABAK and percentage agreement were conducted as a measure of inter-rater reliability (Table 5)

### Testing of validity

**Fig. 4:**
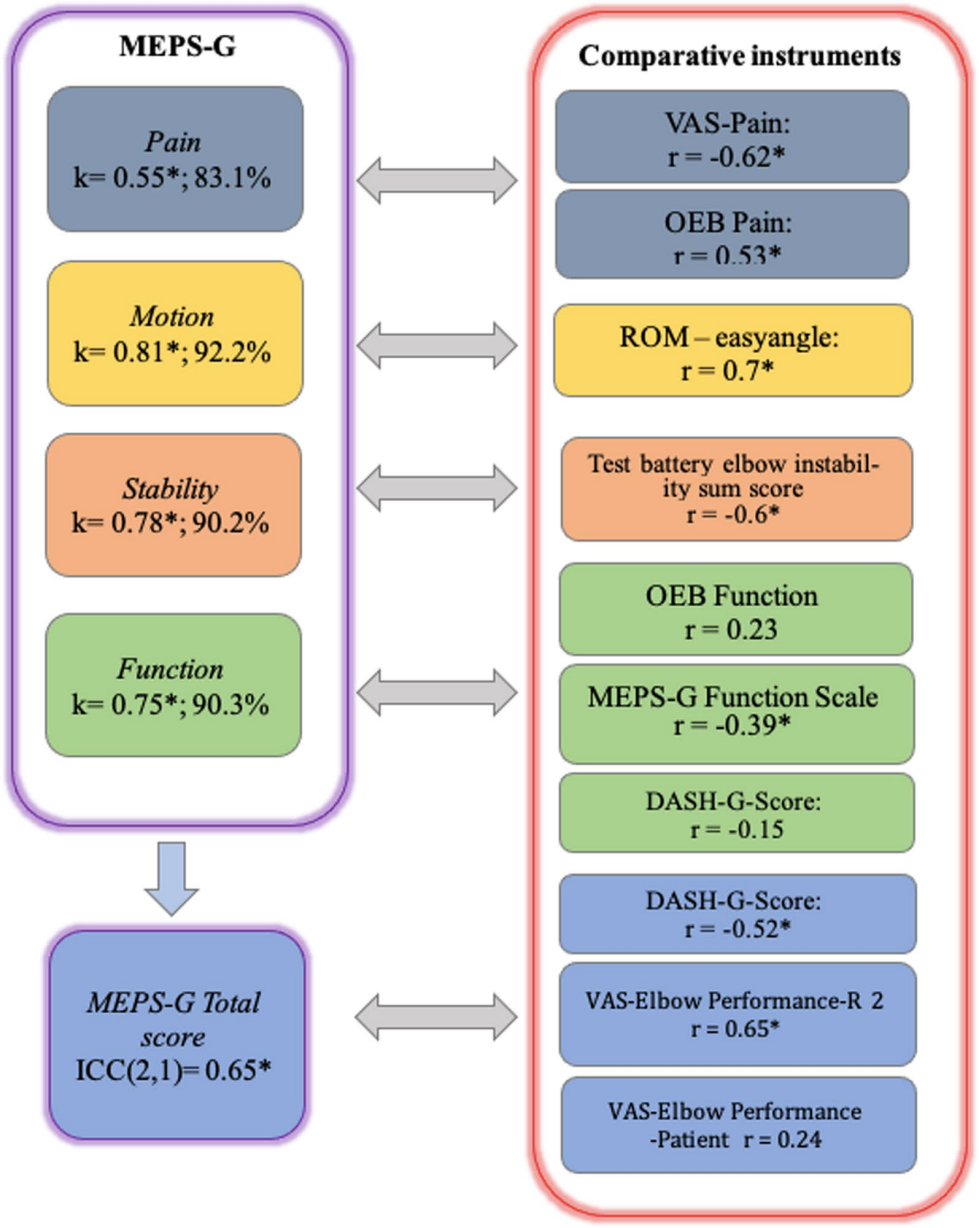
Testing of reliability (violet frame) and validity (red frame) for sum score and each dimension. Abbreviations: % = percentage agreement rater 1 and 2; DASH-G = German Version of Disabilities of the Arm, Shoulder and Hand; ICC = intraclass correlation coefficient; *k* = weighted Kappa (PABAK); MEPS-G = German Version of Mayo Elbow Performance Index; OEB = German Version of the Oxford Elbow Score; *r* = Spearman correlation; R2 = Rater 2; ROM = range of motion; VAS = visual analogue scale. *The correlation is significant at the 0.01 level (two-sided)

The Spearman correlation of the MEPS-G sum score with the DASH-G is *r* = − 0.52 and is statistically significant. The correlation of the VAS-Elbow Performance, estimated by R2, shows a good correlation with the MEPS-G score. The correlation coefficient in relation to VAS-Elbow Performance estimated by the patient, is *r* = 0.24. However, the correlation is not statistically significant (Table 6). The validity of the dimensions is visualized in Fig. 4.

**Table 6:** Criterion and construct validity of the MEPS-G sum score. Spearman’s correlation

Endpoint	MEPS-G R2	VAS-Elbow performance (patient)	VAS-Elbow performance (R2)	DASH-G
MEPS-G R2	1.000			
VAS-Elbow performance (patient)	0.24	1.000		
VAS-Elbow performance (R2)	0.65**	0.32*	1.000	
DASH-G	− 0.52**	− 0.47**	− 0.53**	1.000

*DASH-G* German version of disabilities of the arm, shoulder and hand; *MEPS-G* German Version of Mayo Elbow Performance Index; *R2* Rater 2; *VAS* visual analogue scale

*The correlation is significant at the 0.05 level (bilateral)

**The correlation is significant at the 0.01 level (two-sided)

## Discussion

The original version, in English, of the MEPS has been evaluated and validated [28, 39–41]. Other cultural adaptations have been carried out, saying that the MEPS is a reliable tool for the assessment of various elbow pathologies. However, regarding the few and somewhat outdated studies and the questionable methodological quality, The et al. [25] consider the MEPS as not being adequately validated. The MEPS was developed without adhering to the scientifically sound methodological principles [24]. The determination of the patient-related dimensions as well as the stability is not described in detail and introduce examiner bias.

This is the first study cross-culturally validating the MEPS and evaluating the validity and reliability of the German version (MEPS-G). Based on the evaluation of 57 patients with six investigators, the MEPS-G showed only sufficient inter-rater reliability and validity of the objective dimensions. The patient-based dimensions are, however, a limitation in this study.

### Reliability

The reliability between more than two investigators collecting the MEPS has not been analyzed before. The agreement of the total score of the MEPS-G between Rater 1 and Rater 2 was moderate to good. The more homogeneous group of five surgeons showed a high agreement. During data collection, the patient-based dimensions were asked and the answers entered. This inevitably leads to an interpretation of the statements. The physiotherapist probably interprets the subjects’ descriptions of pain and function differently than the medical professionals. De Boer et al. [39] found an inter-rater reliability of ICC 0.97. However, only two investigators were included in their study. In the present study, the ICC was tested among a group of six investigators with a moderate-to-good agreement, demonstrating sufficient practicability of the MEPS-G in everyday clinical practice. Nevertheless, it might be problematic for research purposes, because an ICC below 0.7 is more sensitive to distortion and measurement errors [35]. The higher agreement of a more homogeneous group can be considered positive in relation to an individual study, but reduces the comparability of studies with the MEPS as an outcome.

Another study evaluated the intra-rater reliability in a test–retest setting with an excellent ICC of 0.89 using the Turkish version of the MEPS (MEPS-T) [41]. As only one investigator collected the MEPS-T, this may have led to more uniform measurements in comparison to our evaluation comprising six investigators. Additionally, that study considered the measurement error calculations (SEM) and minimal detectable change (MED). Both values were lower in our study, with a SEM of 4.1 (compared to 11.42) and a MED of 11 points (vs. 31 points), respectively.

The main weakness of the MEPS-G seems to lie in the assessment of pain. In our study, this dimension had the least agreement between the six raters. Since this dimension influences for approximately 66% the variance of the MEPS-G sum score [42], the survey of this dimension should be conducted with as little distortion as possible. De Boer et al. [39] had the dimensions pain filled in by the patients. They calculated a test–retest reliability of the dimensions of ICC = 0.72–0.85 (*n* = 42). The pain dimension of the MEPS-G and the MEPS in general can be viewed critically, since only its current intensity is questioned. Neither specific situations, such as “under stress” or “at rest,” nor its duration or quality are considered. Since pain is strongly influenced by psychosocial factors and can therefore be perceived to a very different degree within one day or on different days [42], this seems to be the reason why sole inclusion of the current pain intensity in the MEPS generates less reproducible results between raters.

The agreement between the raters in the dimension motion is the highest for the individual dimensions, and can be interpreted as almost excellent. To the best of our knowledge, this is the first investigation to test the inter-rater-reliability of the dimension motion.

In the present study, the agreement between the raters in the dimension stability is relatively high. In contrast, another research group only reported a weak agreement between the raters (*k* = 0.09) [39]. However, only two raters examined 17 patients with rheumatoid arthritis, and anterior–posterior and varus–valgus instability were assessed at 90° of elbow flexion. In addition, the calculation was carried out with Cohen’s Kappa although a calculation with weighted kappa is more appropriate for the ordinally scaled dimension stability.

The inter-rater-reliability of the dimension function is good. Only De Boer et al. [39] described the reliability of this single dimension. Since in that study patients filled in the dimension, this is the test–retest reliability, which they calculated using Spearman rank correlation and which can be interpreted as excellent (*r* = 0.9; *n* = 42).

### Validity

The total score of the MEPS-G can be considered valid. The correlation with the VAS-Elbow Performance R2 is moderate to good, but agreement with the patient’s view appears to be low. Other studies found a slightly higher agreement of the MEPS sum score and a patients administered 5-point Likert scale “overall severity of impairment” [28]. The investigator’s estimation of this 5-point Likert scale also showed good agreement with the MEPS sum score. Schneeberger et al. [43] compared the MEPS sum score with a self-assessment scale SEV and also evidenced a higher agreement than in the present study (*r* = 0.671; correlation according to Pearson). The SEV, unlike the VAS-Elbow Performance, surveys for a numerical value. Furthermore, pain and function are explicitly integrated into the question of the SEV: “What is the overall percent value of your elbow if a completely normal elbow represents 100% and if an elbow with extreme pain and no function represents 0%?”. In addition, in that study the investigators supplemented the MEPS dimension pain with information on activities and pain medication intake “none, mild (no limitation of activity and occasional use of analgesics), moderate (limitation of activity and regular use of analgesics), or severe,” thus distorting the most influential dimension of MEPS [43].

The correlation of the MEPS-G score with DASH-G is fair to moderate and corresponds to the finding of other studies. Turchin et al. [28] were able to demonstrate a correlation of the MEPS sum score with the DASH of *r* = − 0.56. Celik demonstrated a comparable correlation of *r* = − 0.61 [41]. However, unlike Turchin et al. or our study, the dimensions of pain and functions were filled in by the patients in Celik’s study [28, 41].

The validity of the dimension pain can be confirmed as in the MEPS-G, VAS-Pain also only records the current pain intensity. The OEB pain score measures the pain experienced in the past 4 weeks, which can most likely explain the somewhat lower, but still moderate, correlation with the dimension pain and confirms our prior hypothesis. Both Turchin et al. and Celik used VAS-Pain as a comparative tool. However, the VAS-Pain was compared with the MEPS sum score and both showed fair-to-moderate agreement using Pearson–product–moment correlation (*r* = − 0.43; *r* = − 0.53) [28, 41].

As of now, a validation of the dimension stability has not been published. In the cultural adaptation of MEPS into Turkish, Celik added an examination of varus–valgus laxity [41]. However, since a PLRI, for example, is the most common form of elbow instability, the addition of varus–valgus laxity seems inappropriate. Elbow instability is more suitable as a diagnostic or prognostic tool and less suitable for testing the effect of an intervention [44]. Therefore, and given the difficulty of assessing elbow stability, which shows high inter-investigator variance, the authors of this study believe that studies using MEPS as an outcome parameter for elbow stability should be critically interpreted.

This study cannot confirm the validity of the dimension functions. The correlations with the comparative instruments DASH-G, OEB function score and MEPS-G function scale are weaker than previously reported. While the elbow specificity of the MEPS-G functions could be questioned, an observation bias could also be present [28]. Likewise, the very clear prevalence of the response categories could exert an influence on the calculations. If the 5-point Likert Scale MEPS-G function scale is correlated with the comparison instruments, good-to-excellent correlations can be identified. The correlation coefficient according to Spearman is *r* = 0.77 with the DASH-G, and *r* = 0.76 with the OEB function score. The MEPS-G function scale was filled in by the patients themselves. However, unlike the dimension function in MEPS-G, it consists of a 5-point Likert scale and not the binary response format. In the study by De Boer et al. [39], patients filled in the dimension functions and compared it with their objective assessment of elbow functions and also found a weak-to-moderate correlation (*r* = 0.3). As in the present study, that study had the limitation that more than 80% of the study participants had a maximum function sum score of 25. It is recommended to further validate the dimension function. Up to now, the binary queried functions in this dimension do not seem to adequately represent the actual function of an affected elbow. The data of this study indicate that external or objective assessments of the function of the elbow do not correspond to the patients’ perceptions. However, it can be discussed whether a patient’s perspective is desired in the MEPS-G.

### Limitations

The selection of comparative instruments was based on a systematic review of the literature. The PROMs DASH-G and OEB used are sufficiently validated measuring instruments and were also used in other studies evaluating the MEPS. Although all dimensions of MEPS-G were collected from the investigators in this study, the dimensions pain and functions should be validated by means of PROMs, since these two dimensions query patient data. The use of PROMs should evaluate the validity of the latent constructs of functions and elbow performance and also the validity of the transferability of patient-based data collected by an external investigator. Validated measurement procedures were also used as clinical tests, but the tests for elbow instability are not as reliable as, for example, the PROMs used. With the help of the test battery instability with two tests for each form of elbow instability, it should be possible to at least approximate a valid statement regarding an existing elbow instability. In retrospect, the study design can be described as target-oriented. The sample size of at least 50 subjects [34] required for reliability testing was achieved. Randomization of the investigating physicians and the patients on the days of the data collection would have been desirable to present a possible systematic disturbance variable in the judgments of the investigators, but this was not possible given the daily clinical routine and the short collection period.

The aim of the study was to translate the MEPS into German and to test its psychometric properties. Since the MEPS was not originally tested for its quality criteria during its development, this study aimed to make a further contribution to collecting more psychometric properties of the MEPS. The most important findings of the present study were only sufficient reliability and validity of the MEPS-G total score. The MEPS-G total score has a satisfactory inter-rater reliability. However, the reliability coefficient increased the more homogeneous the sample of the investigators was. This indicates a weakness of the score. The reliability of the most important dimension pain is only moderate. The validity of the dimension functions requires further investigation. Additionally, the survey of the dimensions pain, stability and function has a risk of bias. These findings, as well as the critical analysis and comparison with other studies, point that the use of the MEPS in a research context may result problematic.

## Conclusion

The MEPS-G is no robust outcome measure for the determination of elbow performance in patients with elbow pathologies, which is consistent with its English version (MEPS). Using this rating system might lead to invalid results. The authors of this study recommend not to use the MEPS or MEPS-G as an outcome measurement in future studies. Should the MEPS or MEPS-G be used despite these limitations, users should instruct the investigators performing data collection to present the scores results in detail to allow the research results to be interpreted and compared more objectively.
